# Cross-cultural measurement invariance in the satisfaction with food-related life scale in older adults from two developing countries

**DOI:** 10.1186/s12955-017-0687-8

**Published:** 2017-05-30

**Authors:** Berta Schnettler, Edgardo Miranda-Zapata, Germán Lobos, María Lapo, Klaus G. Grunert, Cristian Adasme-Berríos, Clementina Hueche

**Affiliations:** 1 Facultad de Ciencias Agropecuarias y Forestales, Universidad de La Frontera, Temuco, Chile; 2 LICSA, Núcleo Científico y Tecnológico en Ciencias Sociales, Universidad de La Frontera, Temuco, Chile; 3 Facultad de Economía y Negocios, Universidad de Talca, Talca, Chile; 4 Universidad Católica de Santiago de Guayaquil, Guayaquil, Ecuador; 5 MAPP Centre, Aarhus University, Aarhus, Denmark; 6 Facultad de Ciencias Sociales y Económicas, Universidad Católica del Maule, Talca, Chile; 7 Centro de Excelencia en Psicología Económica y del Consumo, Núcleo Científico y Tecnológico en Ciencias Sociales, Universidad de La Frontera, Temuco, Chile

**Keywords:** Aged, Quality of life, Food, Psychometric, Cross-cultural comparison, Developing countries

## Abstract

**Background:**

Nutrition is one of the major determinants of successful aging. The Satisfaction with Food-related Life (SWFL) scale measures a person’s overall assessment regarding their food and eating habits. The SWFL scale has been used in older adult samples across different countries in Europe, Asia and America, however, there are no studies that have evaluated the cross-cultural measurement invariance of the scale in older adult samples. Therefore, we evaluated the measurement invariance of the SWFL scale across older adults from Chile and Ecuador.

**Methods:**

Stratified random sampling was used to recruit a sample of older adults of both genders from Chile (mean age = 71.38, SD = 6.48, range = 60–92) and from Ecuador (mean age = 73.70, SD = 7.45, range = 60–101). Participants reported their levels of satisfaction with food-related life by completing the SWFL scale, which consists of five items grouped into a single dimension. Confirmatory factor analysis (CFA) was used to examine cross-cultural measurement invariance of the SWFL scale.

**Results:**

Results showed that the SWFL scale exhibited partial measurement invariance, with invariance of all factor loadings, invariance in all but one item’s threshold (item 1) and invariance in all items’ uniqueness (residuals), which leads us to conclude that there is a reasonable level of partial measurement invariance for the CFA model of the SWFL scale, when comparing the Chilean and Ecuadorian older adult samples. The lack of invariance in item 1 confirms previous studies with adults and emerging adults in Chile that suggest this item is culture-sensitive. We recommend revising the wording of the first item of the SWFL in order to relate the statement with the person’s life.

**Conclusions:**

The SWFL scale shows partial measurement invariance across older adults from Chile and Ecuador. A 4-item version of the scale (excluding item 1) provides the basis for international comparisons of satisfaction with food-related life in older adults from developing countries in South America.

## Background

According to the World Health Organization “an adult is a person older than 19 years of age unless national law defines a person as being an adult at an earlier age" [1], whereas an “older people are generally defined according to a range of characteristics including: chronological age, change in social role and changes in functional abilities. In high-resourced countries older age is generally defined in relation to retirement from paid employment and receipt of a pension, at 60 or 65 years [2]. Regarding older adults, healthy or successful aging refers to the improvement and preservation of physical, social and mental well-being, independence and quality of life [3]. Life satisfaction is an important concept in the analysis of successful aging [3]. Low satisfaction with life among older adults may produce various adverse outcomes; a recent study even suggests that it can predict dementia [4]. Conversely, older adults with greater life satisfaction have the lowest risk of mortality [5]. Satisfaction with life corresponds to a person’s positive assessment of their life in general, or of particular domains such as family, studies, work, health, friends, free time and food [6]. Regarding the food domain, various authors suggest that nutrition is one of the major determinants of successful aging [7–14], and it is at the same time related with food-related well-being [15] and with overall life satisfaction [16] in older adults.

Thus, we must measure the role of food-related well-being in healthy aging in order to assess and monitor it. The most widely used instrument for this purpose is the Satisfaction with Food-related Life (SWFL) scale, which was developed and validated by Grunert, Dean, Raats, Nielsen and Lumbers in three studies on older adults in eight European countries [17]. The SWFL scale, consisting of five items, measures a person’s overall assessment regarding their food and eating habits [17] and shows satisfactory psychometric properties in samples of older adults [17, 18], adults [19, 20] and young people [21]. The SWFL scale has been used in older adult samples in different countries from Europe [17, 22–24], Asia [15, 25, 26], and South America [18, 27, 28]. In these studies, higher levels of satisfaction with food-related life have been linked with higher levels of life satisfaction [19, 26, 28], greater levels of happiness [18, 27], fewer physical and mental health problems [17, 23, 26, 28] and isolation [26], better perceived health status [28], greater family support [23, 26, 28], healthful eating habits [15, 28], but also with enjoying food and meals [23, 28], higher income and better living circumstances [23], food service quality and quality of life [25], area of residence [15, 28] and gender [24] in older adults.

Although some authors conclude that well-being is not related to age [29], others suggest that life satisfaction is age-sensitive [30]. At the same time, eating habits change throughout a person’s life stages [14, 31, 32], and being older is a predictor of poor food intake that could result in malnutrition problems [14, 32, 33]. Satisfaction with food-related life is likely to change with changes in the environment or the person [14]. Therefore, it could also be expected that SWFL changes over the years, making studying satisfaction with food-related life very relevant at the elderly stage of life. However, the prevalence and types of nutritional problems among the elderly vary according to country, health care setting and local resources [33]. Recent cross-cultural studies have reported differences in the preferences and attitudes towards food and nutrition in older adult samples. Mingioni et al. [34] measured attitudes towards fruits and vegetables using the Health and Taste Attitude Scales (HTAS) questionnaire in older adult samples from Finland, Poland, France, UK and Spain. Allès et al. [9] administered a Food Frequency Questionnaire to register the weekly frequency of consumption of 40 categories of foods and beverages for each of the three main meals in older adult samples from France and Canada. Healy [35] analyzed how older adults from Ireland, the UK, France and Italy differ in terms of food-related practices based on the available data from the household budget survey (HBS). In addition, the first study regarding satisfaction with food-related life using the SWFL scale reported the highest levels in Germany and the UK, and the lowest levels in Poland, Italy and Portugal [17]. These differences may be linked with country resources or level of economic development [33], or they could also be associated with cultural differences across countries.

If we want to investigate how satisfaction with food-related life changes with age, contingent on the cultural differences that exist between countries, we need to assure that the measuring instrument is valid in a cross-cultural setting for people of different age groups. Although the SWFL scale has demonstrated good psychometric properties [17, 18, 20], recent works in which the SWFL was used to predict satisfaction with life [19, 21] showed that two of the SWFL scale items did not work well in the Chilean context. For an adult sample, the first item of the SWFL had to be omitted to obtain an acceptable fit [19]. In a second study with university students, a good fit could only be achieved by eliminating the second item [21]. This suggests that the SWFL could be sensitive to both cultural and age-related differences and that the cross-cultural validity of the SWFL merits more attention.

Studies on the cross-cultural performance of measuring instruments evaluate whether or not the same instrument activates similar perceptive, cognitive and interpretive processes in groups with different cultural backgrounds [36]. A recent study showed that the SWFL scale exhibited strict invariance (equal factor loadings, thresholds and residuals) when comparing Chilean and Spanish undergraduate student samples [37]. However, to our knowledge there are no studies that have evaluated the cross-cultural measurement invariance of the SWFL in older adult samples, even though the scale has been used in various studies conducted in different countries from three continents [15, 17, 22–28]. Therefore, it is important to establish measurement invariance. Without it, we are unable to decipher whether differences across groups in means or correlates of satisfaction with food-related life are true differences or are actually just due to psychometric differences in item responses [38]. Thus, assessing the cross-cultural measurement invariance of the SWFL scale in older adults will enable the creation of common policies to improve satisfaction with food-related life during this stage of life, in which nutrition is a relevant determinant of successful aging [7–14].

Therefore, the present study assesses the measurement invariance of the SWFL scale across two samples of older adults from two developing countries in South America. The South American context is important because increased life expectancy is reflected in the growing older adult population in these countries [25]. It is estimated that the number of people over 65 in Latin America and the Caribbean in 2030 will be double the number registered in 2010, rising from 9.8% to 16.7% of the total population [39]. Generally, there are still few studies that have examined health, nutrition and well-being in older adults from developing countries in South America [40]. In our study, Chile and Ecuador were selected for comparison. Although both are Spanish-speaking countries, they are nations with significant cultural differences. Based on the cultural dimensions of the Hofstede framework, Hofstede and Bond found differences between Chilean and Ecuadorian citizens for power distance, individualism vs. collectivism, masculinity vs. femininity and in the uncertainty avoidance dimension [41]. Furthermore, although feeding and lifestyle patterns in Latin America have been affected during the last decades due to globalization and urbanization processes, there are important differences between the two countries. While in Chile the prevalence of malnutrition is very low, and while it is one of the South American countries with the highest proportion of obese adults (about 30%), Ecuador has a moderately high prevalence of malnutrition [42].

## Methods

### Sample and procedure

The Ethics Committee of the Universidad de Talca approved the study protocol in Chile. The Ethics Committee at the Universidad Católica de Santiago de Guayaquil accepted the study protocol in Ecuador. In both countries, the inclusion criterion for the sample was individuals aged 60 years or older of both genders, without physical (functional) or mental (dementia) disabilities and who were able to sign written informed consent. Only older adults who voluntarily agreed to participate were surveyed and the anonymity of the respondents was protected.

A power analysis was carried out using the G*power 3.1 program [43]. Then, a minimum sample size of 694 participants was set for this study in both countries (Cronbach’s alpha = 0.05, effect size = 0.25, power (1-β) = 0.95, allocation ratio N2/N1 = 1.0).

Stratified random sampling with proportional affixation within each commune was conducted in both countries. The Chilean sample was recruited from 30 communes of the Maule Region of central Chile. The sample was divided into two sub-groups: the first included older adults living in urban areas, and the second older adults living in rural areas. We applied two-stage sampling, stratified by clusters with incidental sub-sampling (casual) and by networks (snowball sampling) within the clusters. As strata, we used the 30 communes of the Maule region, with allocation proportional to the size of the sample population. Within each commune, we performed allocation per cluster, as well as allocation proportional to the sample population size. The stratum resulted as the intersection between commune and cluster. We used nursing homes in the National Registry of Social Organizations for Elderly Persons, part of Chile’s National Service for Elderly Persons (SENAMA), as clusters. We selected the nursing homes in each stratum via simple random sampling, with the “random sample of cases” function in the Statistical Package for Social Sciences (IBM SPSS) v. 23.

The Ecuadorian sample was recruited from the province of Guayas in Ecuador. We again performed a two-stage sampling process, stratified by clusters with incidental sub-sampling (casual) and by networks (snowball sampling) within the clusters. We used 25 communes in the province of Guayas as strata, with allocation proportional to the size of the sample population. Within each commune, the allocation was by cluster, also proportional to sample population size. The stratum resulted as the intersection between commune and cluster. Gerontology centers were used as clusters. The gerontology centers within each stratum were selected by simple random sampling with the “random sample of cases” function in the Statistical Package for Social Sciences (IBM SPSS) v. 23.

Trained interviewers visited the nursing homes in Chile and the gerontology centers in Ecuador and contacted individuals who met the sample inclusion criteria. The interviewers explained the objectives of the study and the strictly confidential treatment of the information obtained. Then, they provided detailed information about the questionnaire and asked the individuals to provide written informed consent if they wished to participate. Given that in Chile the nursing homes registered in SENAMA correspond to gathering spaces for older adults oriented towards improving the aging process, we asked the older adults who agreed to participate if they preferred to answer the questionnaire at the nursing home or in their houses, and the day and time to apply the questionnaire were set according to participant availability. On the other hand, due to the fact that the gerontology centers in Ecuador correspond to long-term residences for older adults, all questionnaires were applied in these places, but following a schedule that was according to the older adults’ availability. Therefore, the Chilean sample only included non-institutionalized older adults, but who were registered in nursing homes affiliated with SENAMA. Meanwhile, in Ecuador, only institutionalized older adults were included. This implies differences in the food of the older adults from both countries, since the Chilean older adults feed themselves in their homes and the Ecuadorian adults participating in this study receive three meals per day at the gerontology center.

The questionnaire was personally administered by the trained interviewers in November 2013 and January 2014 in Chile and in March and July 2015 in Ecuador. The trained interviewers read the questionnaire questions aloud and recorded the participant responses on the paper questionnaires.

### Instrument

As part of a larger questionnaire, older adults reported their levels of satisfaction with food-related life by completing the Satisfaction with Food-related Life (SWFL) scale [17]. It consists of five items grouped into a single dimension: 1. “Food and meals are positive elements”; 2. “I am generally pleased with my food”; 3. “My life in relation to food and meals is close to ideal”; 4. “With regard to food, the conditions of my life are excellent”; 5. “Food and meals give me satisfaction in daily life”. Respondents were asked to indicate their degree of agreement with the statements using a 6-point Likert scale (1: disagree completely; 6: agree completely). A Spanish-language version of the SWFL scale was used in this study, which showed good levels of internal consistency (Cronbach α = 0.85–0.86) in previous studies with older adults in Chile [18, 27] and Ecuador [28]. The score can range from 5 to 30, and higher scores correspond to greater levels of SWFL.

## Data analysis

To analyze measurement invariance of SWFL, we used the software Mplus v. 7.3. Following Forero, Maydeu-Olivares and Gallardo-Pujol [44], the polychoric correlation matrix was used to perform the CFA, using Robust Unweighted Least Squares (ULSMV) as the estimation method, given the ordinal scale of the items.

In order to test the invariance of the factor model in the two samples, configural invariance and measurement invariance (weak invariance, strong invariance and strict invariance) were checked [45]. To determine the achievement of configural invariance, the model Chi-square (χ2), Comparative Fit Index (CFI), Tucker-Lewis Index (TLI), and Root Mean Square Error of Approximation (RMSEA) were used. Criteria for good model fit were a non-significant model Chi-square, CFI > 0.96, TLI > 0.95, and RMSEA <0.06 [46]. An acceptable model fit was considered when CFI > 0.90, TLI > 0.90, and RMSEA <0.08 [47]. However, considering that the Chi-square test is sample size sensitive [48] and is not recommended to compare nested models, the Satorra-Bentler scaled Chi-square [49] for nested models difference (SB χ2) was used. Thus, to establish that measurement invariance is fulfilled, a non-significant Delta of the Satorra-Bentler scaled Chi-square was required. Partial invariance was considered when strong invariance was not achieved. Dimitrov points out that when there is no perfect invariance for specific parameters, but nor is there evidence of their complete inequality, this indicates partial invariance [45]. In this case, the thresholds estimation of one item was freely estimated, thus testing strong partial invariance. To determine the thresholds to be freed in strong partial invariance, a modification index (MI) over 3.84 was considered, choosing first the largest value of MI [45].

## Results

A total of 840 older adults were approached in Chile and 962 in Ecuador. The response rate was 90% in Chile and 84.9% in Ecuador. Therefore, the sample consisted of 756 participants in Chile and 817 in Ecuador. Thus, although the minimum sample size required was 694, we collected data from more participants in both countries based on the expectation of missing data or error responses.

The mean age of the Chilean sample was 71.38 (SD = 6.48, range = 60–92 years), and 66.3% of respondents were females. The mean age of the Ecuadorian sample was 73.70 (SD = 7.45, range = 60–101 years), and 47.5% were female. In this study, the mean SWFL score of all participants was 23.41 (SD = 3.98, range = 7–30) in Ecuador and 23.06 (SD = 3.79, range = 5–30) in Chile.

The SWFL scale was assessed for cultural invariance by starting from the baseline configural invariance followed by invariant factor loadings, invariant thresholds and invariant residuals (Table 1). The χ2 test was significant (χ2 (12) = 35.294, *p* < 0.05) in Model 0, however it is well established that χ2 test is sensitive to the sample size [50]. Therefore, considering the indices of fit, the overall fit of the baseline model was regarded as good (CFI = 0.998; TLI = 0.997; RMSEA = 0.050), which established the configural invariance of the SWFL scale across Chilean and Ecuadorian older adults. This model served as a basis for comparison of the weak or metric measurement invariance model.

**Table 1:** Measurement invariance for SWFL model between Chilean and Ecuadorian older adults’ samples

Model	χ^2^	df	D_SB χ^2^	D_df	p_D_SB χ^2^	RMSEA	CFI	TLI
Model 0 Configural (without invariance)	35.294	12	-	-	-	0.050	0.998	0.997
Model 1 Weak (loadings fixed)	43.474	15	4.651	3	0.199	0.049	0.998	0.997
Model 2 Strong (loadings and thresholds fixed)	63.928	33	33.104	18	0.016	0.035	0.998	0.999
Model 3 Strong partially invariance (loadings and thresholds fixed, with thresholds of item 1 freed)	55.560	32	25.880	17	0.077	0.049	0.998	0.999
Model 4 Strict invariance (loadings, thresholds fixed, with thresholds of item 1 freed and residuals fixed)	51.345	37	3.970	5	0.554	0.022	0.999	0.999

χ2 = Chi square

df = Degrees of freedom

SB χ2 = Satorra-Bentler adjustment of Chi-square

D = Delta between a model and previous model

D_SB χ2 = Delta of Satorra-Bentler adjustment of Chi-square

D_df = Delta of degrees of freedom

p_D_SB χ2 = Statistical significance of Delta of Satorra-Bentler adjustment of Chi-square

RMSEA = Root Mean Square Error of Approximations

CFI = Comparative Fit Index

D_CFI = Delta of CFI

TLI = Tuker-Lewis Index

**Fig. 1:**
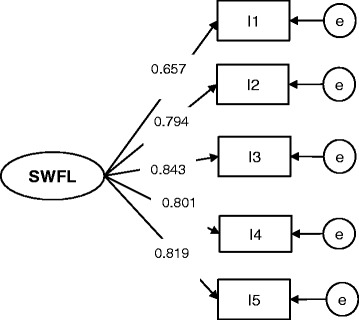
SWFL model for the Chilean older adults’ sample

**Fig. 2:**
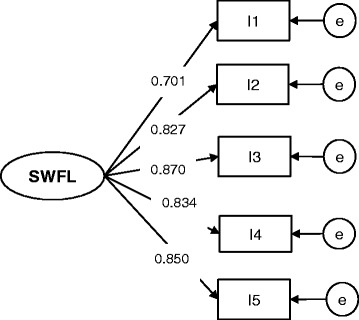
SWFL model for the Ecuadorian older adults’ sample

Then, factor loadings were constrained to be equal across groups to test for weak invariance (Table 1). The SB χ2 difference test between configural invariance (Model 0) and weak invariance (Model 1) models was not significant (D_SB χ2 (3) = 4.651, *p* > 0.05), demonstrating that the weak invariance was supported. Therefore, the factor loadings were invariant across older adults groups (Figs. 1 and 2). Thus, the items on the SWFL scale were related to the latent variable in the same way across the two countries’ older adult samples.

Next, equality of thresholds across groups was imposed on the model to test for strong or scalar invariance (Model 2, Table 1). The SB χ2 difference test between Model 1 and Model 2 was significant (D_SB χ2 (18) = 33.104, *p* < 0.05), indicating that the thresholds are not invariant across the two elderly groups. The examination of the MIs shows the MI value for the thresholds of item 1 is greater than the critical of 3.84, suggesting that this item’s thresholds were not invariant. Thus, Model 2 was modified by releasing the constraint on this item’s thresholds. The resulting modified model was labeled Model 3 (strong partial invariance). The SB χ2 difference test between Model 1 and Model 3 was not significant (D_SB χ2 (17) = 25.880, *p* > 0.05), indicating that strong partial invariance was supported. Therefore, there are invariant thresholds across the two countries, except for the thresholds of one item (item 1).

Next, equality of residuals item variances (uniqueness) across groups was imposed on the model to test for strict invariance (Model 4, Table 1), with thresholds of item 1 freely estimated (Table 1). The SB χ2 difference test between Model 3 and Model 4 was not significant (D_SB χ2 (5) = 3.970, *p* > 0.05), indicating that strict partial invariance was supported. This finding indicates that the item residuals were approximately constant across both older adult groups.

Therefore, it is possible to establish that there is configural invariance and partial measurement invariance, with invariance of all factor loadings, invariance of all but one item thresholds (item 1) and invariance of all items’ uniqueness (residuals), which leads us to conclude that there is a reasonable level of partial measurement invariance for the CFA model of the SWFL scale.

Given that the study of group-differences is valid only when strong measurement invariance is reached, we conducted an independent sample t-test using only items 2–5 to compare mean levels of SWFL scores between older adults from Chile and Ecuador, using the Statistical Package for Social Sciences (IBM SPSS) v. 23. Table 2 shows the mean scores from these four items of the SWFL scale and the scale’s total score in both sub-samples (with a new theoretical range of 6–24). No significant differences were detected in the SWFL mean scores nor in the item mean scores between older adults from Chile and Ecuador (*p* > 0.1).

**Table 2:** Mean score of four items of the Satisfaction with Food Life (SWFL) scale and mean score of the SWFL in Chilean and Ecuadorian older adult’s samples

Variable	Chilean sample		Ecuadorian sample		*P*-value
	Mean	SD	Mean	SD	
Item 2	4.57	0.99	4.62	1.07	0.347
Item 3	4.49	0.99	4.57	1.02	0.133
Item 4	4.51	0.95	4.53	0.98	0.782
Item 5	4.74	0.86	4.78	0.95	0.289
SWFL	18.33	3.18	18.51	3.38	0.278

*P* value correspond to Student’s t-test for related samples (paired) for homogenous variances

Item 2: I am generally pleased with my food

Item 3: My life in relation to food and meals is close to ideal

Item 4: With regard to food, the conditions of my life are excellent

Item 5: Food and meals give me satisfaction in daily life

## Discussion

Successful aging is an important topic for nutrition research, with increasing worldwide interest as the number of older adults continues growing [8, 33]. In fact, aging has been related with an increased risk of malnutrition [7, 8, 10–12, 14, 32, 33], decreased nutrient intake [8, 12, 14], unintentional weight loss and sarcopenia [11, 12, 14], functional disabilities [8, 12, 14], overweight and obesity [8, 32] and increased mortality [11, 12, 14], among others. However, food is not only important to older adults’ physiological well-being, but also contributes to social, cultural and psychological well-being [8], and with overall quality of life [11, 14, 32]. Considering that older adults’ higher levels of satisfaction with food-related life have been associated with greater psychological well-being [17, 18, 23, 26–28], higher social well-being [23, 26, 28], better eating habits and enjoyment of food and meals [15, 23, 28] and higher quality of life [25], the SWFL scale may be a useful tool to detect variables that could improve health-related aspects, social and psychological well-being, successful aging and quality of life in older adults. Considering that nutrition intervention among older adults encloses the potential to promote healthful and more active aging [33], the SWFL scale may be used by researchers, practitioners and health workers to improve older adult quality of life in different parts of the world. In fact, the attractive features of the scale, including its applicability in a wide range of contexts and its brevity, can be particularly harnessed by health workers who work with older adults in order to quickly and simply detect low levels of satisfaction with food-related life and to efficiently respond to improving this aspect and the previously mentioned associated variables.

Although one previous study has assessed cross-cultural invariance of the SWFL [37], this study is the first to explore the cross-cultural measurement invariance of the SWFL scale in older people. Our results show configural invariance of the SWFL scale across older adults (over 60 years old) from Chile and Ecuador. This supports the presence of a single factor model across groups, and indicates that Chilean and Ecuadorian older adults conceptualize satisfaction with food-related life in the same one-dimensional structure. In addition, our results show weak invariance, which indicates that the five items are related to the latent variable in the same way across Chilean and Ecuadorian older adults. However, only partial scalar or strong invariance was supported, as item 1 (*Food and meals are positive elements*) thresholds were not invariant. This means that the groups’ mean differences at the observed level do not reflect the groups’ mean differences at the latent level [51]. This contradicts a previous study that reported strict invariance of the SWFL in undergraduate university students samples from Chile and Spain [37], which may be related to the different ages and cultures of the samples. However, we also note other previous studies using Chilean samples [19, 21], where SWFL was used to predict life satisfaction in adults and university students, and where item 1 had to be omitted in order to obtain an acceptable fit. This suggests that item 1 of the SWFL performs differently in different contexts and hence that scale validity is culture-specific, since item 1 showed a good behavior in the studies conducted to develop and validate the SWFL with samples of older adults in Europe [17]. Based on the previous findings and the lack of invariance in the item 1 thresholds in this study, we suggest that South American and European people interpret the meaning of “food and meals are positive elements” differently, as reported in studies that have assessed the cross-cultural invariance in scales that measure subjective well-being [38, 52]. A possible explanation may be related to the wording of item 1, which refers to food and meals in general and not to the individual’s food-related life, whereas in items 2 to 5, wording is related to the person’s pleasure, their ideal food-related life, their relation between food and life conditions, and individual satisfaction with their food and meal on a daily basis. In fact, it is possible to expect that a person could better assess their satisfaction with food and eating habits, thinking of his/her own food and meals, and not thinking of food and meals in general. Therefore, we recommend revising the wording of the first item of the SWFL in order to relate the statement to the person’s life (e.g. “Food and meals are positive elements in my life”), when the SWFL measurement is applied in samples comprising individuals from South America, and evaluate their psychometric properties including cross-cultural measurement invariance. In fact, some authors recommended that psychometric properties should be routinely evaluated when applied to different samples [53].

Although more research is needed in order to explain the differing interpretation of item 1, we suggest that a 4-item version of the SWFL (items 2–5) would be a valid measure to compare the levels of satisfaction with food-related life in older adults from Chile and Ecuador [6, 38, 52]. While it is possible to expect that the meals received by older adults may influence their levels of satisfaction with food-related life, it is noteworthy that no significant differences were detected between the SWFL scores from both samples, although Chilean participants have their meals at home whereas Ecuadorian participants in this study have their meals at the gerontology centers. In addition, taking into account that strict measurement invariance was supported, the 4-item version of the SWFL scale may also be used to investigate cross-cultural correlations between the levels of satisfaction with food-related life and external variables [51]. Therefore, the similar structure of the 4-item version SWFL scale across these samples provides the basis for meaningful international comparisons of satisfaction with food-related life in developing countries. This would allow health workers and health organizations related to older adults to create common policies to improve food-related well-being and the quality of life of this quickly growing population across the world.

However, it is important to mention that our results are from a comparison of two groups of older adults who speak the same language, from relatively close South American countries. Thus, notwithstanding evidence of cultural differences between Chile and Ecuador [41] and specifically in nutrition related issues [42], our findings may partly be related to cultural similarities between these two countries. Therefore, more research is needed to assess the SWFL’s cross-cultural measurement invariance between countries with larger cultural differences in general and in the food domain, and between countries that speak different languages. In this regard, analyzing SWFL’s cross-cultural measurement invariance considering Western vs Eastern countries, and developing vs developed nations, would be beneficial.

The limitations of this study include: it was conducted only in two countries; both are developing countries; only one geographic area was evaluated in each country. This does not allow us to generalize the results and further research is needed to evaluate the cross-cultural measurement invariance of the SWFL between countries with greater cultural differences. In addition, despite the random sampling methods, there were fewer females in the Ecuadorian sample, and there were proportionally more females in the Chilean sample than in either country’s elderly population (over the age of 60) in Ecuador [50] and Chile [54]. Another study limitation is that questions regarding the medical conditions of each participant, which may affect their diet/food (e.g. current tooth pain, ill-fitting dentures, side effects from medication altering appetite/taste, presence of illness such as cancer/diabetes, etc.), were not included in the questionnaire. These medical conditions may affect participant responses to various items of the scale, including item 1, and their level of satisfaction with-food related life. Therefore, it is considered necessary for future research to take this variable into account since it may have affected participant assessment regarding whether “Food and meals are positive elements”, in the event that one of the sub-samples may have presented a greater number of participants with medical conditions that could affect their diet. All data were self-reported; therefore recall bias or social desirability may have skewed responses, despite the fact that we assured participants that data would be treated as strictly confidential.

Nevertheless, the present study can be considered an advancement in cross-cultural validation of the SWFL for older adults. Our results suggest that SWFL is indeed culture-sensitive, therefore, researchers need to confirm the SWFL scale’s measurement invariance [38, 55] before comparing cross-cultural scores, because item perception may vary in populations from different countries [38].

## Conclusions

The SWFL scale shows configural invariance and partial measurement invariance, with invariance in all factor loadings, invariance in all but one item’s thresholds (item 1) and invariance in all items’ uniqueness (residuals) in older adults aged over 60 years old from Chile and Ecuador. This indicates a reasonable level of partial measurement invariance for the CFA model of the SWFL scale. Nevertheless, a 4-item version of the scale (excluding item 1) provides a basis for meaningful international comparisons of satisfaction with food-related life in older adults from developing countries in South America. This is important considering the growth of the older population in Latin American developing countries and the Caribbean. This would allow the creation of common policies that could improve food-related well-being and older adults’ quality of life in similar countries.

## Abbreviations

CFA: Confirmatory factor analysis
CFI: Comparative fit index
CMA: Center of major adult
MI: Modification index
RMSEA: Root mean square error of approximation
SB χ2: Satorra-Bentler scaled chi-square
SWFL: Satisfaction with Food-related Life
TLI: Tucker-Lewis index
ULSMV: Robust unweighted least squares
χ2: Chi-square
